# Testing a conceptual framework of loneliness, social isolation and health outcomes in older adults

**DOI:** 10.1186/s12877-026-07003-x

**Published:** 2026-02-11

**Authors:** Arunika Pillay, Ke Ning, PV AshaRani, Edimansyah Abdin, Peizhi Wang, Anitha Jeyagurunathan, YunJue Zhang, Rajeswari Sambasivam, Janhavi Ajit Vaingankar, Saleha Binte Shafie, Jianlin Liu, Harish Gopalakrishna Magadi, Fengyuan Yao, Rathi Mahendran, Ng Li Ling, Siow Ann Chong, Mythily Subramaniam

**Affiliations:** 1 Research Division, Institute of Mental Health, Singapore, 539747 Singapore; 2 Saw Swee Hock School of Public Health, National University of Singapore, Singapore, 117549 Singapore; 3 Lee Kong Chian School of Medicine, Singapore, 308232 Singapore; 4 Department of Geriatric Psychiatry, Institute of Mental Health, Singapore, 539747 Singapore; 5 Department of Psychological Medicine, National University of Singapore, Singapore, 119228 Singapore; 6 Department of Psychological Medicine, Changi General Hospital, Singapore, 529889 Singapore

**Keywords:** Social disconnection, Cardiovascular health, Cerebrovascular health, Cognitive function

## Abstract

**Background:**

Loneliness and social isolation are established risk factors for adverse cardiovascular, cerebrovascular and cognitive outcomes in later life, yet the pathways underlying these associations remain underexplored. This study tests the structure of a published framework linking social isolation and loneliness to health outcomes through psychological, behavioural and physiological pathways.

**Methods:**

We analysed cross-sectional data from 1,685 Singaporean adults over the age of 60 from the Wellbeing of the Singapore Elderly study. Path analysis was used to examine the psychological (subsyndromal and syndromal anxiety and depression), behavioural (smoking, alcohol use, poor diet and sedentary lifestyle), and physiological (hypertension, obesity, diabetes and sleep disruption) pathways from social isolation and loneliness to health outcomes (cardiovascular, cerebrovascular and cognitive health).

**Results:**

The final model fit the data well (χ²(60) = 67.62, *p* = .233; CFI = 0.986; TLI = 0.993; RMSEA = 0.009). Loneliness was positively associated with psychological burden (*b* = 0.20, *p* < .001), unhealthy behaviours (*b* = 0.06, *p* = .001), and physiological burden (*b* = 0.06, *p* = .005). Social isolation was positively associated with psychological burden (*b* = 0.07, *p* = .005) and unhealthy behaviours (*b* = 0.14, *p* < .001). Physiological burden was positively associated with cardiovascular disease (*b* = 0.22, *p* < .001) and cerebrovascular conditions (*b* = 0.34, *p* < .001), while unhealthy behaviours were positively associated with cerebrovascular conditions (*b* = 0.21, *p* = .003) and poorer cognitive function (*b* = -0.14, *p* = .004). Social isolation also showed direct associations with poorer cognitive function (*b* = -0.09, *p* = .007) and lower probability of cardiovascular disease (*b* = -0.08, *p* = .014).

**Discussion:**

Social isolation and loneliness are associated with behavioural and physiological factors that increase vulnerability to cardiovascular, cerebrovascular and cognitive conditions. Routine screening and public health strategies to address social disconnection and associated factors can improve wellbeing and reduce healthcare burden. Longitudinal research can clarify causal mechanisms and inform targeted interventions.

**Supplementary Information:**

The online version contains supplementary material available at 10.1186/s12877-026-07003-x.

## Introduction

 Social disconnection, encompassing social isolation and loneliness, is increasingly recognised as a global epidemic [1] with estimated economic and healthcare costs of US$2–25 billion annually [2]. Social isolation refers to an objective lack of social connections while loneliness reflects subjective distress from a perceived gap between desired and actual connections [3]. The two constructs can exist independently yet remain interrelated, each linked to adverse health outcomes via psychological (e.g. depression and anxiety), behavioural (e.g. smoking, alcohol use, diet and activity), and physiological (e.g. metabolic conditions and sleep) pathways [4, 5]. As such, understanding their distinct impacts is critical. Older adults are particularly vulnerable to social disconnection due to reduced mobility, declining health, loss of interpersonal networks, family fragmentation and financial instability [6]. In Singapore, 40% of the population over 60 years report feeling lonely [7], a concern amidst rapid population aging and growing evidence linking social disconnection to poor health [5].

Evidence consistently links social disconnection to cardiovascular disease, cerebrovascular events such as stroke and transient ischemic attacks (TIA), and adverse cognitive outcomes. A meta-analysis reported a 29% increase in risk of developing cardiovascular disease and greater recurrence of cardiovascular events [8] alongside longitudinal links to mortality and heart failure [9, 10] among socially disconnected individuals. Similarly, disconnection has been shown to increase risk of first-time stroke by 32% [11] and recurrent stroke by 40% [12], with experimental and animal studies further indicating improved recovery with greater pre-ischemic social interaction [13], and worse injury with isolation [14]. Although, findings on cognitive effects are inconsistent, longitudinal evidence demonstrates that loneliness is associated with a 41% higher risk of cognitive impairment [15, 16] while meta-analytic evidence suggests links between social disconnection and mild cognitive impairment [17]. Notably, while social disconnection is often conceptualised as contributing to poor health, it may also arise from illness (e.g. stroke) or disability, supporting the plausibility of bidirectional effects [18, 19].

The relationship between social disconnection and adverse health outcomes may be explained through psychological, behavioural and physiological pathways [5, 18]. Loneliness is robustly linked to depression and anxiety [20, 21] which may partially explain the link to cardiovascular disease and related mortality [22, 23]. While the associations between social isolation and depression and anxiety are less established, the combination of social isolation and psychological conditions heighten the risk for subsequent cardiovascular events [21]. Behaviourally, lonely and isolated individuals were more likely to smoke, consume alcohol, make poor dietary choices and adopt a sedentary lifestyle [24, 25]. Health behaviours have been found to mediate the association between loneliness and health outcomes [26]. Physiologically, loneliness and social isolation are associated with metabolic conditions such as hypertension [27], obesity and type 2 diabetes [28], as well as reduced sleep quality and duration [20], all of which elevate the risk for poor cardiovascular, cerebrovascular and cognitive outcomes.

However, few studies have sought to empirically evaluate these pathways within an integrated framework, with most focusing on isolated associations or single pathways between social disconnection and health outcomes. The present study aims to address this by examining the structural validity of the framework proposed by Cené and colleagues (adapted from Hodgson et al.) [5, 18] using path analysis on cross-sectional data from Singaporean older adults. The framework was selected for its comprehensive, testable structure that synthesises prior conceptual work [18] and specifies directional paths between constructs. Given indicator availability, we modelled observed composite variables for each domain, allowing for a parsimonious representation of the framework’s pathways. To this end, we proposed the following hypotheses:

- H1: Social disconnection (i.e., loneliness and social isolation) will be directly associated with adverse psychological, behavioural, and physiological factors.
- H2: Psychological, behavioural, and physiological factors will be directly associated with poorer cardiovascular, cerebrovascular, and cognitive outcomes.
- H3: The associations between social disconnection and health outcomes will be partially accounted for by these intermediate factors, consistent with the pathways proposed in the original framework.
- H4: Given the bidirectional influences posited by the original model, an alternate model specifying reverse pathways will also demonstrate good fit.

While the adapted model implies mediation [5], the present study focused on testing the structural relationships without estimating indirect effects due to the risk of bias with cross-sectional estimates [29]. Structural testing of the model is critical even in the absence of mediation analysis, as it permits examination of whether the hypothesised relationships between key constructs hold when considered simultaneously within a broader network. Although, the cross-sectional nature of our data precludes causal inference, this study provides initial validation of the model’s organisation and assumptions, offering foundational evidence for further longitudinal investigations. To our knowledge, this is the first empirical structural test of a social disconnection framework in an Asian context, that simultaneously models three intermediary domains (psychological, behavioural, physiological) and three health outcomes (cardiovascular, cerebrovascular, and cognitive). In addition, we evaluated an alternate directional model to account for potential bidirectional influences, and discuss implications for screening and upstream prevention in ageing populations.

## Methods

This study utilised data from the Wellbeing of the Singapore Elderly (WiSE, 2023), a cross-sectional study replicating the methodology of the original WiSE [30]. Data were collected from March 2022 to September 2023.

### Sampling strategy

A disproportionate stratified sampling method was used to randomly select older adults in Singapore, that were over the age of 60, from an administrative database that consisted of names, ages and addresses of citizens and permanent residents. Participants were stratified into three age groups (60–74, 75–84, 85 years and above) and four ethnic groups (Chinese, Malay, Indian, and Others), yielding 12 strata. Individuals were then randomly sampled, with oversampling of older age and minority ethnic groups to ensure sufficient representation. Full details are described in a previous publication [31].

The original study targeted a sample size of 2000, assuming 10% dementia prevalence, 2% margin of error, 80% power, and α = 0.05, with a design effect of 1.934 due to oversampling. For the present analyses, a post-hoc analysis revealed that with a sample of 1685, 49 degrees of freedom, and α = 0.05, power to detect inadequate model fit (i.e., RMSEA > = 0.06) was 1.00.

### Participants

**Fig. 1:**
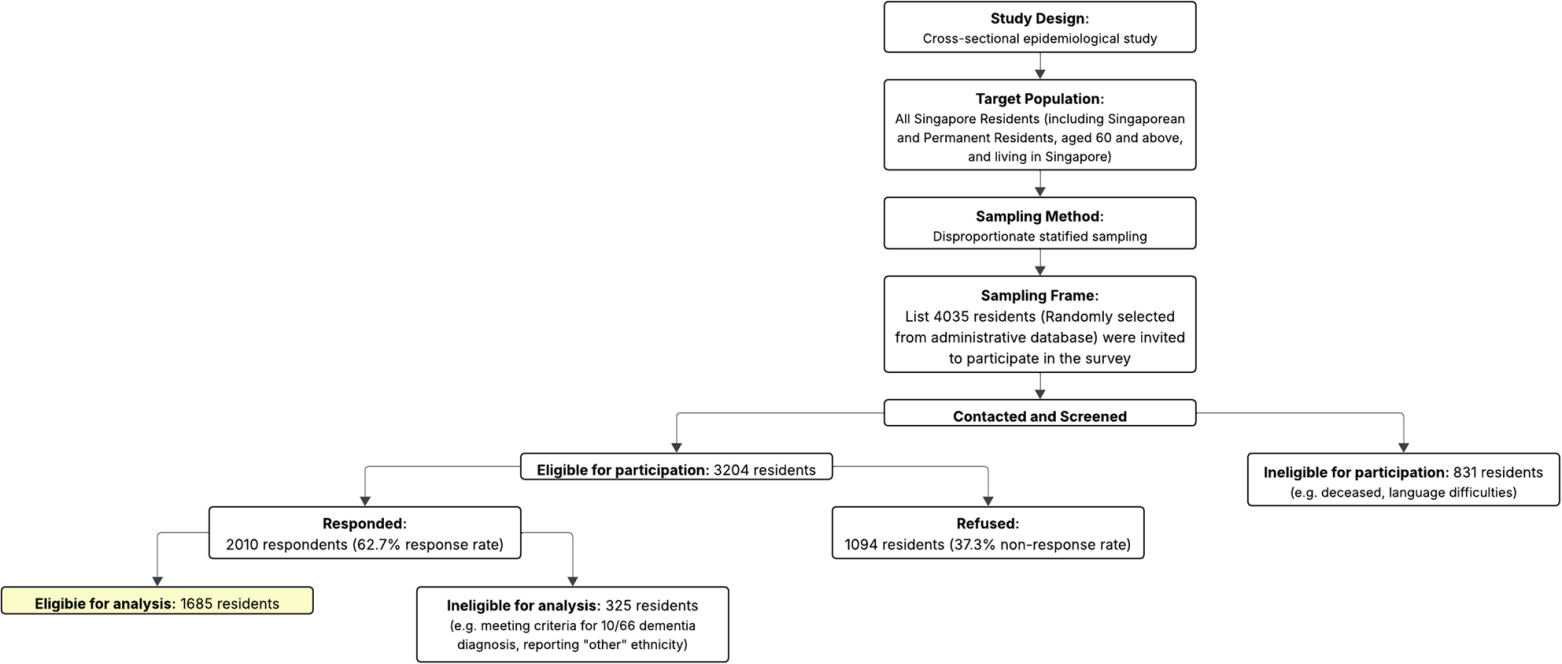
Recruitment flow diagram

Older adults were invited via residential mail, followed by house visits by trained interviewers. Invitations were provided in English and the respondent’s spoken language (i.e. Chinese, Malay or Tamil). Participants were not eligible for participation if they were (i) unable to provide informed consent and did not have a suitable legal representative, (ii) not currently residing in Singapore during the study. Figure 1 depicts the participant recruitment flow.

### Measures

Measures were limited to those within the WiSE study dataset. Variables were derived based on the availability of measures and their relevance to the constructs of interest.

#### Social isolation

A social isolation index was computed guided by previously published methods (e.g. [32]), by assigning 1 point for each of the following conditions met, and 0 if not met: (a) was unmarried, widowed or divorced/separated, (b) was living alone, c-e) had less than monthly contact with children, family members or friends, f) did not participate in community activities, social groups, clubs, lectures or religious meetings. Total scores (0–6) were used as a continuous measure, with higher scores indicating greater isolation. For item-level distribution of the social isolation index, please see Supplementary Table S1.

#### Loneliness

Loneliness was measured using the 6-item *De Jong Gierveld Loneliness Scale*, a self-report instrument capturing emotional and social loneliness [33]. Responses were coded as 1 (lonely) or 0 (not lonely), summed into a continuous score from 0 to 6, with higher scores indicating greater loneliness. The scale has demonstrated good psychometric properties across cultures [33] and acceptable internal consistency (Cronbach’s α = 0.61) within our sample [34], comparable to prior validations across European and Asian populations (typically α = 0.61–0.81) [35].

#### Cardiovascular conditions

The participants’ history of cardiovascular conditions was determined by asking the following question: “Have you ever been told by a doctor that you had heart trouble?” (yes/no) from an adapted version of the *Health Status Questionnaire*.

#### Brain health

The conceptual model defines brain health as encompassing cerebrovascular conditions and cognitive function [5]. However, in our analysis, these were modelled as separate observed variables. Dementia (as diagnosed using the 10/66 algorithm) was also excluded due to high missingness on the loneliness scale among affected participants.

##### Cerebrovascular conditions

Two *Health Status Questionnaire* items assessed cerebrovascular history, including strokes and transient ischemic attacks (TIA). Items included “have you ever had a stroke that needed medical attention?” (yes/no) and “have you ever developed sudden weakness of a limb, loss of speech, or partial blindness that improved within one day” (yes/no). For analysis, these items were combined into a binary variable with ‘none’ and ‘one or more conditions’ as response categories.

##### Cognitive function

Cognitive performance was measured using the *Community Screening Instrument for Dementia (CSI’D)*, comprising of the Consortium to Establish a Registry for Alzheimer’s Dementia (CERAD) verbal fluency task and modified 10-word recall tasks. An item-weighted global score (COGSCORE; 0–32) was used. Only COGSCORE was retained due to its objectivity while other 10/66 components (e.g. RELSCORE) were excluded [31]. It is important to note that the cognitive performance assessed primarily reflects verbal learning, memory, and language-based fluency. It does not capture broader domains such as processing speed, visuospatial ability, or complex attention.

#### Psychological factors

Anxiety and depression were assessed using the Geriatric Mental State (GMS) interview and classified with the Automated Geriatric Examination for Computer Assisted Taxonomy (AGECAT) system [31]. AGECAT assigns levels indicating non-case (0), subsyndromal symptoms (levels 1–2), or syndromal/clinical disorder (level 3 or more) for each condition. For the present analysis, each condition (depression and anxiety) was dichotomised such that AGECAT levels 1 or greater were coded as ‘1’ (presence of subsyndromal/syndromal disorder) and level 0 as ‘0’ (absence). The two dichotomous indicators were then summed to create an ordinal composite with three categories, ‘none’, ‘one condition’, or ‘two conditions’. The prevalence for anxiety and depression are reported in Supplementary table S2.

#### Physiological factors

This included three metabolic indicators (obesity, hypertension, diabetes) and sleep disturbance. Obesity was defined as BMI ≥ 30 kg/m². History of hypertension and diabetes were assessed using the respective items *Health Status Questionnaire* items “Have you ever been told by a doctor that you have high blood pressure?” (Yes/No) and “Have you ever been told by a doctor that you had diabetes?” (Yes/No). Sleep disturbance was measured using one GMS-AGECAT item: “Trouble with sleep or recent change in pattern” (0 = none, 1 = presence of disturbance). For analysis, these were combined into an ordinal variable indicating ‘none’, ‘one condition’, ‘two conditions’ or ‘three or more conditions’ reflecting increasing physiological burden.

#### Behavioural factors

Health behaviours (alcohol use, tobacco use, diet, physical activity) were assessed via the *Health Status Questionnaire*. Alcohol use was assessed using item “After the age of 60, what is/was the most you would drink in a week?” and coded as 1 (currently drink) or 0 (does not currently drink). Tobacco use was assessed by item ““Has there ever been a period when you smoked cigarettes, cigars or a pipe, chewing tobacco, beedi or snuff nearly everyday? Do you still use tobacco regularly?” and coded as 1 (current smoker) or 0 (past/never). Dietary behaviours were measured by item “How many servings of fruit and vegetables have you eaten over the last 3 days?” Responses were dichotomised as ‘healthy’ (5 or more servings daily) or ‘unhealthy’ (less than 5 servings daily). Physical activity was assessed using the item “Taking into account both work and leisure, would you say that you are”: “very physically active”, “fairly physically active”, “not very physically active” or “not at all physically active”. Responses were dichotomised with the former two options as ‘active’ and the latter two as ‘not active’. An ordinal variable captured the number of unhealthy behaviours: “none”, “one”, “two” or “three or more” [25].

The original composites of behavioural factors and physiological conditions can range from 0 to 4. However, only eight participants had all four unhealthy behaviours, and only nine had all four physiological conditions. To ensure sufficient category count for analysis, we combined scores of three and four into a single ‘three or more’ category. The derived composites were not treated as continuous, as the limited distribution and small category counts at the upper end would not support a reliable continuous specification. Prevalence and missingness for each behavioural and physiological item are reported in Supplementary Table S2.

#### Covariates

Sociodemographic covariates (Table 1) included age, sex (male/female), ethnicity (Chinese, Indian, Malay), monthly personal income (no income, 1–1000 SGD, 1000–3000 SGD and 3000 SGD or above), education (no formal education, below primary, primary, secondary, tertiary) and employment (currently working, not working).

**Table 1:** Sample characteristics of participants included in the analysis

	**Categories**	**Mean**	**SD**	**Missing %**
Continuous				
Sociodemographics				
Age	-	72.4	8.3	0.1%
Exposures				
Loneliness	-	1.5	1.5	1.2%
Social Isolation	-	1.6	1.3	0.4%
Outcomes				
Cognitive function	-	28.7	2.3	0.0%
		**n**	**%**	**Missing %**
Categorical				
Sociodemographics				
Gender	Male	790	46.9%	0.0%
	Female	895	53.1%	
Monthly income	No income	247	14.7%	0.5%
	$1 - $1000	673	40.1%	
	$1001 - $3000	575	34.3%	
	Above $3000	182	10.9%	
Ethnicity	Chinese	612	36.3%	0.0%
	Malay	568	33.7%	
	Indian	505	30.0%	
	Others	0	0.0%	
Education	No formal education	122	7.3%	0.2%
	Below primary	301	17.9%	
	Primary	502	29.9%	
	Secondary	498	29.6%	
	Tertiary	258	15.3%	
Employment	Currently working	623	37.1%	0.2%
	Not working	1058	62.9%	
Intermediate variables				
Psychological	None	1212	71.9%	0.0%
	One condition	314	18.6%	
	Two conditions	159	9.4%	
Behavioural	None	66	4.0%	2.1%
	One unhealthy behaviour	981	59.5%	
	Two unhealthy behaviours	521	31.6%	
	Three or more unhealthy behaviours	81	4.9%	
Physiological	None	330	25.5%	23.3%
	One condition	486	37.6%	
	Two conditions	364	28.2%	
	Three or more conditions	113	8.7%	
Outcomes				
Cardiovascular conditions	No	1381	82.3%	0.4%
	Yes	297	17.7%	
Cerebrovascular conditions	None	1566	93.3%	0.4%
	One or more conditions	113	6.7%	

*SD* Standard deviation, *n* = frequency, *%* proportion of the sample, *Missing %* proportion of missingness, *SSD *Subsyndromal depression, *$* Singapore dollars (SGD)

### Statistical analysis

Path analyses were conducted in R (v4.4.1) using the lavaan package (v0.6–19) [36]. Of 2010 total participants, 1,685 were included in the final sample. Exclusions were based on 10/66 dementia diagnosis [31], or reporting “other” ethnicity. Most variables had ≤ 5% of missing data (Supplementary Table S2), except physiological factors (23.3%) due to missing BMI measurements (see Tables 1 and Supplementary Table S2) as height/weight measurements were not obtained for all participants. Because this missingness was related to logistical constraints rather than participant characteristics, we consider MCAR to be plausible. Missing data was handled using pairwise deletion.

**Fig. 2:**
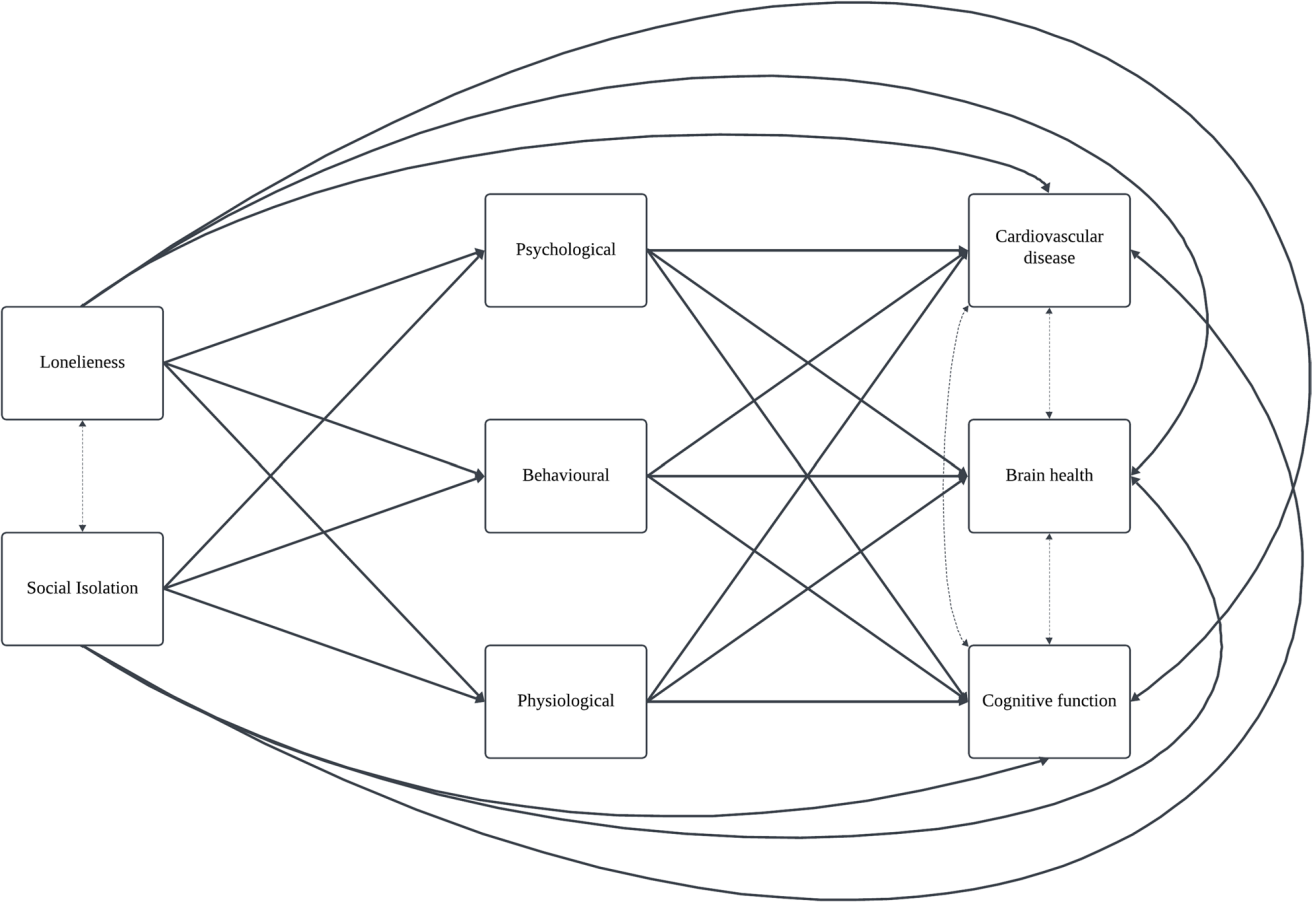
Model to be tested Solid arrows indicate direct relationships. Dotted arrows represent covariances. Covariates (age, sex, ethnicity, income, education, employment) will be adjusted for in analysis

Path analysis with observed variables was used to evaluate the framework [5], which posits that social disconnection affects health outcomes through psychological, behavioural and physiological pathways. Loneliness and social isolation were modelled as exposures; cardiovascular conditions, cerebrovascular conditions and cognitive function as outcomes; and psychological, behavioural and physiological factors as intermediate variables linking them (Fig. 2). The variables were modelled as observed, due to limited empirical support for the latent constructs. Covariances were added between exposures, and between outcomes. Age, gender, income, ethnicity, education and employment were included as covariates.

Given the presence of categorical indicators, models were estimated using the robust weighted least squares estimator (WLSMV). Model fit was assessed using the Comparative Fit Index (CFI), Tucker-Lewis Index (TLI) and Root Mean Square Error of Approximation (RMSEA) with 95% confidence intervals. A good model fit was indicated by CFI ≥ 0.95, TLI ≥ 0.95 and RMSEA ≤ 0.06. Chi-square (χ²) values are also reported but were not used as the primary criterion for evaluating model fit due to its sensitivity to large sample sizes. Given the cross-sectional design and literature suggesting bidirectional associations [18], we also tested the model with reversed directionality with health outcomes as exposures and social disconnection as outcomes.

Solid arrows indicate direct relationships. Dotted arrows represent covariances. Covariates (age, sex, ethnicity, income, education, employment) will be adjusted for in analysis.

## Results

### Sample characteristics

Socio-demographic characteristics of the sample are presented in Table 1. The sample (*N* = 1,685) had a mean age of 72.4 years (*SD* = 8.3), with 790 men and 895 women. 36.3% of the participants were Chinese, 33.7% Malay and 30% Indian. Most had secondary education or below (84.7%) and a monthly income of ≤ SGD 1000 (54.8%). Mean loneliness and social isolation scores were 1.5 (*SD* = 1.5) and 1.6 (*SD* = 1.3), respectively. Most participants did not have any psychological condition (71.9%), 18.6% had one condition and 9.4% had two conditions. A majority (59.5%) engaged in one unhealthy behaviour, 31.6% in two and 4.9% in three or more. Metabolic and sleep conditions affected 37.6% of the sample. Diagnosed heart disease was reported by 17.7%, cerebrovascular conditions by 7.3% and mean cognitive score was 28.7 (*SD =* 2.3).

Using the dichotomisation threshold (score ≥ 2) applied in sensitivity analyses, 43.4% of participants were classified as lonely and 48.0% as socially isolated.

### Correlation analysis

Table 2 summarises correlations between key study variables. Loneliness was positively associated with psychological (*r* =.22, *p* <.001) and behavioural factors (*r* =.12, *p* <.001), and negatively associated with cognitive function (*r* = −.05, *p* <.05). Social isolation was positively associated with psychological (*r* =.11, *p* <.001) and behavioural factors (*r* =.14, *p* <.001), and negatively associated with cardiovascular health (*r* = −.05, *p* <.05) and cognitive function (*r* = −.19, *p* <.001). Psychological, behavioural and physiological factors were positively associated with all three health outcomes.

**Table 2:** Correlations between key study variables

Variables	1	2	3	4	5	6	7	8
1. Loneliness	1							
2. Social Isolation	0.15***	1						
3. Psychological factors	0.22***	0.11***	1					
4. Behavioural factors	0.12***	0.14***	0.19***	1				
5. Physiological factors	0.03	0.03	0.25***	0.07**	1			
6. Cardiovascular conditions	0.03	−0.05*	0.11***	0.1***	0.17***	1		
7. Cognitive Function	−0.05*	−0.19***	−0.07**	−0.14***	−0.11***	−0.03	1	
8. Cerebrovascular conditions	0.05	0.02	0.07*	0.11***	0.14***	0.09***	−0.01	1

Note. Pearson’s correlation was used. Significance levels: *p <.05, **p <.01, ***p <.001

### Path analysis

**Fig. 3:**
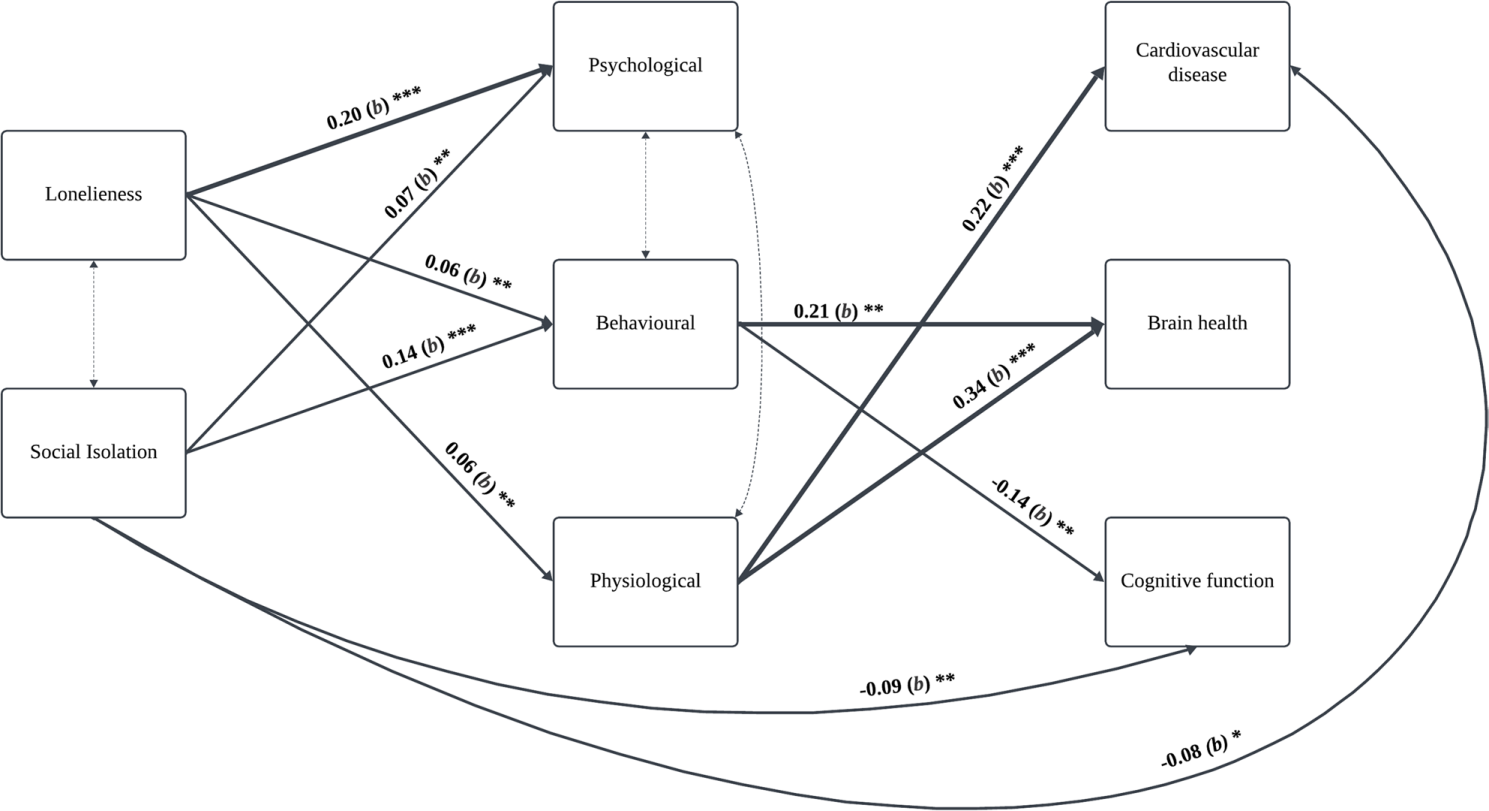
Final model Solid arrows indicate direct relationships. Bold lines indicate b ≥ 0.2. Dotted arrows represent covariances. Covariates (age, sex, ethnicity, income, education, employment) were adjusted in analysis. Only significant paths are presented but all paths were modelled. *p <.05 **p **≤**.01 ***p **≤**.001

We tested the proposed conceptual model [5] (Fig. 3). The initial model, which included covariances between predictors (social isolation and loneliness) and between health outcomes, showed moderate fit: χ²(3) = 166.37, *p* <.001, CFI = 0.69, TLI = −1.85, RMSEA = 0.18. In addition to modification indices, we drew on prior literature outlining the interdependent and bidirectional nature of the intermediary domains. Psychological distress is known to influence, and be influenced by, metabolic and sleep-related dysregulation [37], and similarly has reciprocal relationships with health behaviours such as smoking, diet and physical activity [38]. Although these interrelations are not depicted in the original framework, they are theoretically consistent with the broader literature. As such, we added covariances between psychological, physiological and behavioural factors, which substantially improved model fit: (χ² (1) = 7.50, *p* <.01, CFI = 0.99, TLI = 0.66, RMSEA = 0.062). Further improvement was achieved by removing covariances between outcome variables, consistent with the view that health outcomes share risk factors rather than direct links χ²(4) = 9.88, *p* <.05, CFI = 0.99, TLI = 0.92, RMSEA = 0.03 [39]. Finally, omitting non-significant covariate paths improved TLI: χ²(49) = 67.62, *p* >.05, CFI = 0.99, TLI = 0.99, RMSEA = 0.01. Categorical covariates were removed using Wald tests [40].

Path coefficients are provided in Table 3 and discussed below.

**Table 3:** Path coefficients for associations between social disconnection and health outcomes

Pairs of Variables		b (95% CI)
Exposure	Intermediate variable	
Loneliness	Psychological	0.20 (0.16, 0.24)***
	Behavioural	0.06 (0.02, 0.10)**
	Physiological	0.06 (0.02, 0.09)**
Social Isolation	Psychological	0.07 (0.02, 0.12)**
	Behavioural	0.14 (0.10, 0.19)***
	Physiological	0.02 (−0.02, 0.07)
Exposure/Intermediate variable	Outcome	
Loneliness	Cardiovascular conditions	0.01 (−0.04, 0.07)
Social isolation		−0.08(−0.15, −0.02)*
Psychological		0.12 (0.00, 0.25)
Behavioural		0.09 (−0.01, 0.18)
Physiological		0.22 (0.11, 0.33)***
Loneliness	Cerebrovascular conditions	0.04 (−0.03, 0.11)
Social isolation		−0.01 (−0.09, 0.08)
Psychological		−0.01 (−0.15, 0.13)
Behavioural		0.21 (0.07, 0.36)**
Physiological		0.34 (0.20, 0.48)***
Loneliness	Cognitive function (Continuous)	−0.04 (−0.10, 0.02)
Social isolation		−0.09 (−0.16, −0.03)**
Psychological		−0.11 (−0.23, 0.01)
Behavioural		−0.14 (−0.23, −0.04)**
Physiological		0.04 (−0.09, 0.17)

* *p* <.05 **p ≤.01 ***p ≤.001, *b* = Unstandardised regression coefficient; CI = confidence interval

#### Psychological factors

Both loneliness (*b* = 0.20, 95% CI [0.16, 0.24], *p* <.001) and social isolation (*b* = 0.07, 95% CI [0.02, 0.12], *p* =.005) were significantly associated with greater number of psychological conditions (i.e. anxiety and depression). However, psychological factors were not significantly linked to greater cardiovascular disease risk, cerebrovascular conditions, or lower cognitive function scores.

#### Physiological factors

Loneliness (*b* = 0.06, 95% CI [0.02, 0.09], *p* =.005), but not social isolation (*b* = 0.02, 95% CI [−0.02, 0.07], *p* =.34) was significantly associated with greater physiological burden (i.e. metabolic and sleep dysregulation). Increased physiological burden was associated with greater risk for cardiovascular (*b* = 0.22, 95% CI [0.11, 0.33], *p* <.001) and cerebrovascular conditions (*b* = 0.34, 95% CI [0.20, 0.48], *p* <.001), but not cognitive score.

#### Health behaviours

Loneliness (*b* = 0.06, 95% CI [0.02, 0.10], *p* =.001) and social isolation (*b* = 0.14, 95% CI [0.10, 0.19], *p* <.001) were associated with more unhealthy behaviours (drinking, smoking, poor diet and inactivity), which were linked to increased cerebrovascular risk (*b* = 0.21, 95% CI [0.07, 0.36], *p* =.003) and lower cognitive scores (*b* = −0.14, *SE* = 0.05, *p* =.004). Unhealthy behaviours were not significantly related to cardiovascular conditions.

#### Direct Effects

Social isolation showed a direct negative association with cardiovascular conditions (*b* = − 0.08, 95% CI [− 0.15, − 0.02], *p* =.014) and cognitive score (*b* = − 0.09, 95% CI [− 0.16, − 0.03], *p* =.007), but not cerebrovascular conditions. Loneliness had no direct effect on any health outcomes.

### Results of the alternate model

We reversed the model direction, treating health outcomes as exposures and social disconnection as dependent variables via the intermediate pathways. Covariances were included between (i) psychological factors and health behaviours (ii) psychological and physiological factors and (iii) social isolation and loneliness. Model fit was good χ²(4) = 8.56, *p* >.05, CFI = 0.99, TLI = 0.94, RMSEA = 0.03. Further non-significant covariates were removed and model fit improved χ²(56) = 55.70, *p* >.49, CFI = 1.00, TLI = 1.00, RMSEA = 0.00 and the pattern of associations was similar to the main model (See Supplementary Table S5 for path coefficients).

### Sensitivity analyses

Due to the skewed distribution of loneliness and social isolation, we dichotomised them using a cut-off of 2 and above [41]. Loneliness was only significantly associated with psychological and behavioural factors, while social isolation was significantly associated with all three intermediary factors. Intermediary variables were also linked to health outcomes in a similar pattern to the main analysis. However, while the direct association between social isolation and cognitive score persisted, the association with cardiovascular disease was no longer significant. Overall, these findings support the key pathways identified in the main analysis (Supplementary Table S3).

A secondary sensitivity analysis addressed missing physiological data arising from BMI (23.3% missing due to absent height/weight measurements). We recomputed the physiological composite excluding BMI and re-estimated the path analysis using the revised variable. Results demonstrated the same pattern of significant pathways as the main model, with no changes in significance of associations. This suggests that missing BMI data did not materially influence the structural relationships observed in the primary analysis (Supplementary Table S4).

## Discussion

This study used path analysis to empirically test the structure of a conceptual framework linking components of social disconnection (loneliness and social isolation) to cardiovascular and brain health outcomes through psychological, physiological and behavioural pathways. To account for bidirectional associations between the components, the model was also tested in the reverse direction.

The findings reported here mostly support the conceptual model [5] as applied to a sample of older adults from Singapore. The final model demonstrated good fit with loneliness and social isolation showing associations with greater psychological conditions and unhealthy behaviours. Psychological factors were not linked to any health outcomes in our sample, conflicting with the conceptual model and our initial hypotheses. Unhealthy behaviours were primarily associated with cognitive decline and cerebrovascular risk as expected. Only loneliness, not social isolation, was linked to physiological factors, which were associated with greater risk of cardiovascular and cerebrovascular conditions. As expected, social isolation was also directly linked to greater cognitive impairment. However, more notably, social isolation exhibited a direct, negative relationship with cardiovascular disease, suggesting that socially isolated individuals were less likely to have been diagnosed with a heart condition. These findings are discussed further below in light of relevant literature.

The associations observed between loneliness, social isolation, depression and anxiety are mostly consistent with prior research. Loneliness has repeatedly been shown to predict greater depressive and anxious symptoms [21]. Similarly, social isolation has also been linked to psychological distress, with some studies finding that the effect attenuates when loneliness is accounted for [42]. However, for our sample, both loneliness and social isolation were linked to greater symptoms of distress. Unexpectedly, though, psychological factors were not associated with cardiovascular, cerebrovascular or cognitive outcomes in the final model. This contrasts earlier work showing robust links between depression, anxiety and increased cardiometabolic and cognitive risk [43, 44]. One possible explanation is that much of the variance typically attributed to psychological distress may have been absorbed by the behavioural and physiological pathways in our model, both of which were associated with health outcomes. This interpretation is consistent with cohort evidence indicating that the cardiometabolic consequences of depression is largely mediated through unhealthy lifestyle behaviours and pathophysiological dysregulation, including autonomic imbalance, HPA-axis activation, metabolic dysfunction and pro-inflammatory responses, rather than direct effects on disease risk [45]. These findings align with broader work showing that the harmful effects of distress are generally less marked than behavioural and metabolic risk factors and often act as triggers in individuals with existing vascular burden [46].

Loneliness, but not social isolation, was also linked to having more metabolic conditions such as hypertension, diabetes and obesity, as well as greater sleep disturbances. This is in line with prior research showing that loneliness is associated with inflammation, HPA axis dysregulation, altered cortisol levels and proteomic signatures linked to metabolic dysfunction [47, 48]. Moreover, we found a significant relationship between physiological factors, cardiovascular disease and cerebrovascular disease. This is well established in the literature with several prospective studies finding metabolic conditions and impaired sleep to be significant risk factors [49–51]. In contrast, no association between physiological factors and cognitive impairment was found despite established links in previous literature [52]. This may suggest that the effect of social disconnection on cognitive function may be better explained through behavioural pathways. It is also important to note that the exclusion of participants with dementia likely reduced variability in cognitive performance, which may have limited our ability to detect associations with upstream metabolic or sleep-related factors. Additionally, our cognitive outcome reflects performance on verbal memory and fluency tasks rather than broader domains such as executive or visuospatial functioning. As such, interpretations regarding “cognitive impairment” pertain specifically to the memory- and language-based abilities captured by the CSI’D COGSCORE.

The number of unhealthy behaviours that participants engaged in was significantly associated with both loneliness and social isolation. Individuals that were socially disconnected were more likely to engage in unhealthy behaviours including drinking alcohol, smoking, eating poorly and neglecting physical activity. This was in turn associated with a higher risk of cerebrovascular disease and cognitive impairment. These findings are in line with previous research [25, 53]. Surprisingly, unhealthy behaviours were not associated with cardiovascular disease in our sample. This contradicts a significant body of literature and may reflect measurement limitations. For instance, the analysis considered the number of unhealthy behaviours rather than their frequency or intensity. However, previous research has indicated that individuals with three or more lifestyle risk behaviours had significantly higher odds of cardiovascular disease [54]. A further limitation may be that cardiovascular disease was measured retrospectively while health behaviours were measured as current behaviours. The lack of association may be attributed to potential changes in participants’ unhealthy behaviours following their diagnosis. This is supported by studies finding greatest changes in unhealthy behaviours (e.g. smoking cessation) following heart disease diagnoses [55].

An additional unexpected finding was the negative direct association that was observed between social isolation and cardiovascular disease, suggesting that socially isolated individuals were less likely to report a history of cardiovascular disease. One possible explanation is that socially isolated individuals may utilise healthcare resources less, and that the negative association reflects underdiagnosis rather than a true protective effect. This is consistent with previous findings that among Singaporean adults aged 60 years and over, a smaller social network was significantly associated with lower odds of physician visits [56]. To further explore this possibility, we conducted a brief post-hoc check, which showed a small negative correlation between social isolation and having visited a primary-care provider in the past three months (*r* = −.07, *p* =.01) and a small positive correlation between recent primary-care use and cardiovascular disease (*r* =.08, *p* =.04). Although these correlations are in the expected directions, their magnitudes are very small, indicating that healthcare utilisation differences are unlikely to fully explain the negative path. Moreover, a sensitivity analysis using a dichotomised measure of social isolation no longer shower a negative association, suggesting that the original findings may have been spurious or unstable.

Finally, social isolation was also directly linked to greater cognitive impairment, consistent with previous literature. A lack of social interaction has been observed to negatively impact memory and reasoning and is linked to cognitive decline [57]. Interestingly, loneliness was not directly associated with any of the health outcomes, suggesting that its impact on cardiovascular and brain health may be primarily through the aforementioned pathways.

This study has notable strengths, in that it empirically tests the structure of a previously untested conceptual framework and applies path analysis to explore complex interrelationships. The study also uses data from older adults, a population that is particularly vulnerable to social disconnection, to understand its impact on critical health outcomes. However, there are important limitations to be acknowledged. Self-report measures were used instead of physiological measures which led to increased missingness in height and weight data, and may have introduced additional biases due to inaccurate recall and subjective interpretation of items. Dichotomisation of variables may have attenuated true associations by reducing variability, or in some cases exaggerated effects by imposing artificial thresholds which can impact the interpretation of the main and sensitivity analyses. Additionally, several intermediary constructs were operationalised using observed composite variables, which may mix heterogeneous indicators within each domain. As observed scores do not adjust for measurement error, some associations may have been attenuated; future work should consider latent-variable models with multiple indicators per construct to allow for clearer representation of each pathway. Moreover, the use of cross-sectional data impedes causal inference, limiting our understanding of whether social disconnection drives health outcomes or vice versa. The model was tested in the reverse direction due to the bidirectional nature of the variables which showed good fit with similar patterns of associations as the final model, reinforcing that causality and direction of impact cannot be inferred from our findings. Finally, our key outcome variables were measured retrospectively (e.g. past cardiovascular and stroke diagnoses), while loneliness and social isolation were measured as current status. This may have introduced temporal inconsistencies, further limiting interpretation of our findings as the onset of heart and brain conditions may have preceded the individual’s feelings of loneliness and social isolation. These limitations highlight the need for future research employing longitudinal designs and time-lagged analyses to establish clearer temporal relationships between social disconnection and health outcomes. Future WiSE waves could be considered for cross-lagged panel models for social disconnection and psychological distress, and time-varying or joint models to evaluate behavioural and physiological pathways.

The conceptual framework and our findings have important implications for public health and clinical interventions. Given that social disconnection is associated with multiple health risks in older populations, the framework can inform the development of holistic interventions such as primary care screening for social disconnection, group-based social engagement programs, and behavioural health referrals to yield significant heart and brain health benefits. In practice, this could take the form of a primary-care screening packet pairing brief loneliness and isolation measures with assessments of depression, sleep and cardiometabolic risk, followed by social prescribing to evidence-based community programs. Our findings also point to specific leverage points for intervention. Unhealthy behaviours emerged as a key intermediary pathway, suggesting that integrating behavioural counselling into community or primary-care settings may help to mitigate downstream cerebrovascular and cognitive risks. Similarly, the observed links with physiological burden underscore the importance of routine monitoring and management of metabolic and sleep-related conditions among socially disconnected older adults. On a policy level, public health and social prescription programmes emphasising social engagement could be integrated into national healthy aging strategies to reduce the healthcare and economic burden of social disconnection [18]. Future research could extend our findings by applying the current framework to other vulnerable populations, broadening our understanding of potential variations in risk mechanisms and intervention needs.

## Conclusions

This study provides empirical support for the conceptual model that delineates the pathways through which loneliness and social isolation affects cardiovascular, cerebrovascular and cognitive health [5]. Loneliness was associated with psychological, physiological and behavioural factors which may increase risk of heart disease, ischemia and cognitive impairment. Social isolation on the other hand was only associated with psychological and behavioural factors, increasing the risk for cognitive impairment and cerebrovascular conditions. Moreover, there was a negative association with cardiovascular disease, suggesting underdiagnosis or healthcare avoidance among socially isolated older adults. While the structure of the conceptual model was mostly supported, our results revealed that the alternate model was also supported, highlighting the need for longitudinal research to clarify causal mechanisms. Overall, our findings support the development of upstream interventions that enhance well-being and reduce the societal and healthcare costs associated with social disconnection among older adults.

## Supplementary Information

Supplementary Material 1.

Supplementary Material 2.

## Data Availability

The datasets analysed during the current study are not publicly available due institutional restrictions but are available from the corresponding author on reasonable request.
