# Supplementary material for: Testing a conceptual framework of loneliness, social isolation and health outcomes in older adults
Source: BMC Geriatr. 2026 Feb 11;26:364. doi: 10.1186/s12877-026-07003-x (PMC12997808; doi:10.1186/s12877-026-07003-x)
Supplement: Supplementary file 1 — Supplementary Material 1. [file 12877_2026_7003_MOESM1_ESM.docx]

S1. Item distribution of the Social Isolation Index.

|  |  | **No** | | |  | **Yes** | | |  |  |
| --- | --- | --- | --- | --- | --- | --- | --- | --- | --- | --- |
|  |  | *n* |  | *%* |  | *n* |  | *%* |  | Missingness *(%)* |
| **Social Isolation Items** |  |  |  |  |  |  |  |  |  |  |
| Living alone |  | 1516 |  | 90.0 |  | 169 |  | 10.0 |  | 0.0 |
| Less than monthly contact with families |  | 1444 |  | 85.9 |  | 237 |  | 14.1 |  | 0.2 |
| Less than monthly contact with friends |  | 1051 |  | 62.6 |  | 629 |  | 37.4 |  | 0.3 |
| Less than monthly contact with neighbours |  | 1224 |  | 72.8 |  | 457 |  | 27.2 |  | 0.2 |
| Did not participate in community activities, social groups, clubs, lectures or religious meetings |  | 1163 |  | 69.1 |  | 519 |  | 30.9 |  | 0.2 |
| Unmarried, widowed or divorced/separated |  | 1050 |  | 62.5 |  | 631 |  | 37.5 |  | 0.2 |

**Note**. The Social Isolation Index was derived from six dichotomous indicators reflecting structural and functional aspects of social connectedness. Scores ranged from 0–6, with higher scores indicating greater isolation.
n= frequency; % = proportion of sample; Missingness = proportion of missing responses.

S2. Item distributions of psychological, behavioural and physiological factors.

|  |  | No |  |  |  | Yes |  |  |  |  |
| --- | --- | --- | --- | --- | --- | --- | --- | --- | --- | --- |
|  |  | *n* |  | *%* |  | *n* |  | *%* |  | Missingness *(%)* |
| **Psychological factors** |  |  |  |  |  |  |  |  |  |  |
| Anxiety |  | 1351 |  | 80.2 |  | 334 |  | 19.8 |  | 0.0 |
| Depression |  | 1387 |  | 82.3 |  | 298 |  | 17.7 |  | 0.0 |
| **Behavioural factors** |  |  |  |  |  |  |  |  |  |  |
| Smoking currently |  | 1495 |  | 89.0 |  | 184 |  | 11.0 |  | 0.4 |
| Drinking currently |  | 1493 |  | 90.3 |  | 160 |  | 9.7 |  | 1.9 |
| Less than 5 servings of vegetables daily |  | 89 |  | 5.3 |  | 1587 |  | 94.7 |  | 0.5 |
| Physically inactive |  | 1302 |  | 77.6 |  | 376 |  | 22.4 |  | 0.4 |
| **Physiological factors** |  |  |  |  |  |  |  |  |  |  |
| Diabetes |  | 1104 |  | 65.8 |  | 575 |  | 34.2 |  | 0.4 |
| Hypertension |  | 670 |  | 39.9 |  | 1008 |  | 60.1 |  | 0.4 |
| Obesity |  | 1095 |  | 84.7 |  | 198 |  | 15.3 |  | 23.3 |
| Sleep disturbance |  | 1448 |  | 86.4 |  | 228 |  | 13.6 |  | 0.5 |

**Note.** Behavioural factors included four dichotomous indicators (current smoking, current alcohol use, <5 servings of vegetables/fruits daily, and physical inactivity). Physiological factors included diabetes, hypertension, obesity, and sleep disturbance. Each item was coded as 0 = No and 1 = Yes prior to creating composite indices. Higher scores indicate greater behavioural or physiological burden.
n = frequency; % = proportion of sample; Missingness = proportion of missing responses.

S3. Path coefficients for sensitivity analysis using dichotomised (score ≥ 2) loneliness and social isolation.

| **Pairs of Variables** |  |  |  |
| --- | --- | --- | --- |
| **Exposure** | **Intermediate variable** |  | ***b* (95% CI)** |
| Loneliness | Psychological |  | 0.30 (0.22, 0.39)*** |
|  | Behavioural |  | 0.07 (0.00, 0.14)* |
|  | Physiological |  | 0.06 (-0.02, 0.13) |
|  |  |  |  |
| Social Isolation | Psychological |  | 0.09 (0.01, 0.17)* |
|  | Behavioural |  | 0.21 (0.13, 0.28)*** |
|  | Physiological |  | 0.08 (0.00, 0.15)* |
| **Exposure/ Intermediate variable** | **Outcome** |  |  |
| Loneliness | Cardiovascular conditions |  | 0.03 (-0.07, 0.14) |
| Social isolation |  |  | -0.09 (-0.19, 0.01) |
| Psychological |  |  | 0.12 (-0.01, 0.24) |
| Behavioural |  |  | 0.09 (-0.01, 0.18) |
| Physiological |  |  | 0.23 (0.12, 0.34)*** |
|  |  |  |  |
| Loneliness | Cerebrovascular conditions |  | 0.10 (-0.03, 0.24) |
| Social isolation |  |  | 0.02 (-0.11, 0.16) |
| Psychological |  |  | -0.03 (-0.17, 0.12) |
| Behavioural |  |  | 0.21 (0.06, 0.35)** |
| Physiological |  |  | 0.35 (0.20, 0.49)*** |
|  |  |  |  |
|  |  |  |  |
| Loneliness | Cognitive function (Continuous) |  | -0.11 (-0.23, 0.01) |
| Social isolation |  |  | -0.17 (-0.28, -0.07)** |
| Psychological |  |  | -0.08 (-0.21, 0.04) |
| Behavioural |  |  | -0.13 (-0.23, -0.04)** |
| Physiological |  |  | 0.04 (-0.09, 0.17) |
|  |  |  |  |

* p<.05 **p ≤.01 ***p≤.001, *b* = Unstandardised regression coefficient, CI = confidence interval

S4. Path coefficients for sensitivity analysis excluding BMI from physiological factors.

| **Pairs of Variables** |  |  |  |
| --- | --- | --- | --- |
| **Exposure** | **Intermediate variable** |  | ***b* (95% CI)** |
| Loneliness | Psychological |  | 0.20 (0.16, 0.24)*** |
|  | Behavioural |  | 0.06 (0.03, 0.10)*** |
|  | Physiological |  | 0.07 (0.04, 0.10)*** |
|  |  |  |  |
| Social Isolation | Psychological |  | 0.07 (0.02, 0.12)** |
|  | Behavioural |  | 0.14 (0.10, 0.19)*** |
|  | Physiological |  | 0.01 (-0.03, 0.06) |
| **Exposure/ Intermediate variable** | **Outcome** |  |  |
| Loneliness | Cardiovascular conditions |  | 0.01 (-0.04, 0.06) |
| Social isolation |  |  | -0.08 (-0.14, -0.01)* |
| Psychological |  |  | 0.11 (-0.01, 0.23) |
| Behavioural |  |  | 0.09 (-0.01, 0.19) |
| Physiological |  |  | 0.24 (0.14, 0.34)*** |
|  |  |  |  |
| Loneliness | Cerebrovascular conditions |  | 0.03 (-0.04, 0.10) |
| Social isolation |  |  | -0.01 (-0.09, 0.08) |
| Psychological |  |  | -0.02 (-0.16, 0.12) |
| Behavioural |  |  | 0.22 (0.08, 0.36)** |
| Physiological |  |  | 0.34 (0.22, 0.46)*** |
|  |  |  |  |
|  |  |  |  |
| Loneliness | Cognitive function (Continuous) |  | -0.04 (-0.10, 0.02) |
| Social isolation |  |  | -0.09 (-0.16, -0.03)** |
| Psychological |  |  | -0.10 (-0.22, 0.02) |
| Behavioural |  |  | -0.14 (-0.23, -0.04)** |
| Physiological |  |  | 0.03 (-0.08, 0.14) |
|  |  |  |  |

* p<.05 **p ≤.01 ***p≤.001, *b* = Unstandardised regression coefficient, CI = confidence interval.

S5. Path coefficients for reverse-direction model

| **Pairs of Variables** |  |  |  |  |
| --- | --- | --- | --- | --- |
| **Exposure** |  | **Intermediate variable** |  | ***b* (95% CI)** |
| **Categorical** |  |  |  |  |
| Cardiovascular conditions |  | Psychological |  | 0.23 (0.13, 0.33)*** |
|  |  | Behavioural |  | 0.12 (0.05, 0.20)** |
|  |  | Physiological |  | 0.28 (0.18, 0.37)*** |
|  |  |  |  |  |
| Cerebrovascular conditions |  | Psychological |  | 0.20 (0.09, 0.31)*** |
|  |  | Behavioural |  | 0.20 (0.10, 0.31)*** |
|  |  | Physiological |  | 0.37 (0.24, 0.49)*** |
|  |  |  |  |  |
|  |  |  |  |  |
| Cognitive function (Continuous) |  | Psychological |  | -0.04 (-0.08, -0.01)* |
|  |  | Behavioural |  | -0.07 (-0.11, -0.04)*** |
|  |  | Physiological |  | -0.01 (-0.05, 0.03) |
|  |  |  |  |  |
|  |  |  |  |  |
| **Exposure / Intermediate variable** |  | **Outcome** |  |  |
| Cardiovascular conditions |  | Loneliness |  | 0.02 (-0.09, 0.13) |
| Cerebrovascular conditions |  |  |  | 0.03 (-0.11, 0.18) |
| Cognitive function (Continuous) |  |  |  | -0.03 (-0.07, 0.01) |
| Psychological |  |  |  | 0.40 (0.30, 0.50)*** |
| Behavioural |  |  |  | 0.01 (-0.08, 0.10) |
| Physiological |  |  |  | -0.02 (-0.12, 0.08) |
|  |  |  |  |  |
| Cardiovascular conditions |  | Social isolation |  | -0.13 (-0.23, -0.03)* |
| Cerebrovascular conditions |  |  |  | 0.00 (-0.14, 0.14) |
| Cognitive function (Continuous) |  |  |  | -0.05 (-0.09, -0.02)** |
| Psychological |  |  |  | 0.11 (0.03, 0.20)* |
| Behavioural |  |  |  | 0.18 (0.10, 0.25)*** |
| Physiological |  |  |  | 0.00 (-0.10, 0.09) |

* p<.05 **p ≤.01 ***p≤.001, *b* = Unstandardised regression coefficient, CI = confidence interval.
