# Supplementary material for: Testing a conceptual framework of loneliness, social isolation and health outcomes in older adults
Source: BMC Geriatr. 2026 Feb 11;26:364. doi: 10.1186/s12877-026-07003-x (PMC12997808; doi:10.1186/s12877-026-07003-x)
Supplement: Supplementary file 2 — Supplementary Material 2. [file 12877_2026_7003_MOESM2_ESM.docx]

**Supplementary Table S2.** Missingness Pattern Table

|  | **Psychological** | **Cognitive Function** | | **Brain Health** | | **Social Isolation** | **Cardiovascular Disease** | | **Loneliness** | | | **Behavioural** | | **Physiological** |  |
| --- | --- | --- | --- | --- | --- | --- | --- | --- | --- | --- | --- | --- | --- | --- | --- |
| 1263 |  |  | |  | |  |  | |  | | |  | |  | 0 |
| 368 |  |  | |  | |  |  | |  | | |  | |  | 1 |
| 27 |  |  | |  | |  |  | |  | | |  | |  | 1 |
| 1 |  |  | |  | |  |  | |  | | |  | |  | 2 |
| 1 |  |  | |  | |  |  | |  | | |  | |  | 1 |
| 12 |  |  | |  | |  |  | |  | | |  | |  | 2 |
| 2 |  |  | |  | |  |  | |  | | |  | |  | 3 |
| 1 |  |  | |  | |  |  | |  | | |  | |  | 1 |
| 1 |  |  | |  | |  |  | |  | | |  | |  | 1 |
| 3 |  |  | |  | |  |  | |  | | |  | |  | 2 |
| 3 |  |  | |  | |  |  | |  | | |  | |  | 5 |
| 3 |  |  | |  | |  |  | |  | | |  | |  | 6 |
|  | 0 | 0 | 6 | | 7 | | | 7 | | 21 | 36 | | 392 | | 469 |

| Complete |  |
| --- | --- |
| Missing |  |
